# Dynamical systems analysis applied to working memory data

**DOI:** 10.3389/fpsyg.2014.00687

**Published:** 2014-07-03

**Authors:** Fidan Gasimova, Alexander Robitzsch, Oliver Wilhelm, Steven M. Boker, Yueqin Hu, Gizem Hülür

**Affiliations:** ^1^Department of Psychology, Ulm UniversityUlm, Germany; ^2^Federal Institute for Education Research, Innovation and Development of the Austrian Schooling System (BIFIE Salzburg)Salzburg, Austria; ^3^Department of Psychology, University of VirginiaCharlottesville, VA, USA; ^4^Department of Psychology, Texas State UniversitySan Marcos, TX, USA; ^5^Department of Psychology, Humboldt UniversityBerlin, Germany

**Keywords:** dynamical systems analysis, intensive longitudinal data, intraindividual variability, B-spline imputation, simulation study

## Abstract

In the present paper we investigate weekly fluctuations in the working memory capacity (WMC) assessed over a period of 2 years. We use dynamical system analysis, specifically a second order linear differential equation, to model weekly variability in WMC in a sample of 112 9th graders. In our longitudinal data we use a B-spline imputation method to deal with missing data. The results show a significant negative frequency parameter in the data, indicating a cyclical pattern in weekly memory updating performance across time. We use a multilevel modeling approach to capture individual differences in model parameters and find that a higher initial performance level and a slower improvement at the MU task is associated with a slower frequency of oscillation. Additionally, we conduct a simulation study examining the analysis procedure's performance using different numbers of B-spline knots and values of time delay embedding dimensions. Results show that the number of knots in the B-spline imputation influence accuracy more than the number of embedding dimensions.

## Introduction

The aim of the present study is to investigate intraindividual variability in a measure of cognitive performance using the dynamical systems modeling approach. Nesselroade (1990a) distinguishes between intraindividual changes, meaning long-term change that occurs on macro time scales, and intraindividual variability, meaning short-term change that occurs on micro scales. Ram and Gerstorf (2009) conceptualize two types of intraindividual variability. Firstly, net intraindividual variability, which is characterized by change that is not systematically ordered in time; the second type is time-structured intraindividual variability, which is systematically ordered in time. Various researchers have emphasized the importance of interpretation of the short-term intraindividual variability. Salthouse et al. (2006) reported results of the within-person variability in cognitive performance. Nesselroade and Salthouse (2004) investigated the relationship between age and short-term intraindividual variability using perceptual motor performance. As a measure of intraindividual variability they used the intraindividual standard deviation (ISD). In the present study we focus on intraindividual variability in weekly achievement in a memory updating task (Oberauer et al., 2000, 2003; Schmiedek et al., 2009) in 9th graders over a period of 2 years. As shown in Oberauer et al. (2000) memory updating is a reliable indicator of working memory. We first introduce the methodological perspective of week to week within-person variability in cognitive performance over 2 years, where we focus on dealing with missing data while applying dynamical systems analysis. The second aspect describes how the week to week within-person structured variability can be predicted by covariates.

The most established and widely used methods to investigate intraindividual change and variability of intensive longitudinal data are structural equation modeling (SEM; Bollen, 1989), hierarchical linear modeling and the multilevel approach (Laird and Ware, 1982; Raudenbush and Bryk, 2002). In order to study intraindividual variability over a short period of time, Wang et al. (2012) proposed an autoregressive model focused on investigating the amplitude of fluctuation and the temporal dependency. We aim to apply a different approach to analyze intensive longitudinal data in order to explore intraindividual variability: the dynamical systems modeling approach (Boker, 2001). Deboeck et al. (2009) highlighted that the methods based on ISD and variance are likely to be insufficient to explore intraindividual variability. They suggest dynamical methods are better able to describe how individuals vary with respect to time. Bermúdez (2010) has given an introduction on why dynamical systems analysis should be used to model cognitive abilities. Statistical approaches based on traditional linear models are not efficient to explore non-linear dependencies in data. The basic aims of dynamical systems modeling are the investigation of the change in repeated observations, and how rapidly these observations change over time (Boker and Bisconti, 2006). Boker and Bisconti (2006) concur that many phenomena in nature can be viewed as dynamical systems. Furthermore, Smith and Thelen (2003) emphasized the usefulness of the dynamic systems technique for developmental psychologists, in order to conceptualize developmental change. Differential equations have the ability to describe a system that changes over time. Theoretical aspects of differential equations for analyzing the behavior of dynamical systems are given in Soong (1973). Oravecz et al. (2009) proposed a modeling tool for longitudinal data based on a solution of the Ornstein-Uhlenbeck process, which is a first-order stochastic differential equation (SDE). Voelkle and Oud (2013) introduced a continuous time model for longitudinal data that solves the problem of different time intervals. Their approach uses SDEs to transform the continuous time model into discrete time. In the literature the SDEs and ordinary differential equations (ODE) based approaches have been discussed by Oud and Folmer (2011) and Steele and Ferrer (2011). Oud and Folmer (2011) proposed modeling a damped linear oscillation with stochastic dynamical systems using the Exact Discrete Model (EDM; Bergstrom, 1988). Alternatively, Steele and Ferrer (2011) used the Latent Differential Equation (LDE; Boker et al., 2004) method, a technique based on ODEs. As pointed out by Steele and Ferrer (2011) the ODE and SDE based approaches distinguish themselves mainly in the error term. Both strategies have their advantages and drawbacks. The approach we adopt in the present study is expressed using an ordinary linear second-order differential equation. Dynamical systems may be realized either in discrete or in continuous time (Nowak and Lewenstein, 1994). Linea differential equations do not necessarily result in linear trajectories (Boker and Bisconti, 2006). The requirements for applying dynamical systems analysis are: repeated observations of each individual to estimate within-person variation, at least three observations and equally/unequally spaced time intervals between measurements (Boker et al., 2010a). As pointed out by Nesselroade and Salthouse (2004), at least three occasions are needed to provide the accurate comparisons of individual differences in within-person variability. Von Oertzen and Boker (2010) found that when using time delay embedding for analysis of intensive longitudinal data, the precision of parameter estimates describing intraindividual fluctuation increases. There are also different methods to investigate the oscillatory pattern in longitudinal data. To analyze the oscillation processes, an autoregressive model can be used in case of equally spaced data. Voelkle and Oud (2013) proposed a continuous time model with person-specific time intervals within and between individuals for oscillating and non-oscillating processes. The disadvantage of their proposed method is that it operates only with fixed effects, without providing random effects to account for individual differences. As noted in Hu et al. (2014) and Boker and Nesselroade (2002), in contrast to the damped linear oscillation model an autoregressive model cannot tackle the phase problem (phases are often not synchronized among individuals).

## Method

### Damped linear oscillator model

One way to analyze time-structured intraindividual variability is a damped linear oscillator model (DLO; Boker, 2001). The DLO model is based on derivatives of a univariate time series, and is expressed as a linear combination of the displacement *X*_*t*_ its first order derivative (velocity) $\dot{X}$_*t*_ and the second order derivative (acceleration) $\overset{..}{X}$_*t*_.

(1) $${\ddot{X}}_{t}=\eta X_{t}+\zeta {\dot{X}}_{t}$$

Where *X*_*t*_ represents the displacement from equilibrium at time *t*, in our case represented by the detrended longitudinal data, $\dot{X}$_*t*_ and $\overset{..}{X}$_*t*_ represent the first and second order derivatives of the displacement *X*_*t*_ with respect to time *t*, and η and ζ parameters are the frequency and dampening coefficients. With η, ζ < 0, the linear oscillator is called damped.

The period of oscillation indicates how long one cycle lasts, and is defined as

(2) $$\lambda_{p}=\frac{2\pi }{\sqrt{-\left(\eta +\zeta^{2}/4\right)}}$$

The solution to the second-order differential equation Equation (1) is given as $X_{t}=A_{0}\sin\left(t\sqrt{-\left(\eta +\zeta^{2}/4\right)}+\delta \right)$. *A*_0_ is the amplitudeand δ is the phase of oscillation at *t* = 0, defined in the interval from 0 to 2π. Boker and Ghisletta (2001) introduced the multilevel approach of a linear oscillator as a random coefficient model, which allows for person-specific η and ζ parameters. Steele and Ferrer (2011) explored emotion self-regulation in couples using a damped linear oscillator. The time-delay embedding method (TDE) is used to estimate derivatives of time series by constructing short sections drawn from long time series (Boker et al., 2004; Boker, 2007). There are several ways to estimate derivatives of time series including Latent Differential Equations (LDE; Boker et al., 2004), Local Linear Approximation (LLA; Boker and Nesselroade, 2002), Generalized Local Linear Approximation of Derivatives (GLLA; Boker et al., 2009) and Generalized Orthogonal Local Derivatives (GOLD; Deboeck, 2010). The LDE approach uses the SEM framework with a three-factor model, whereby the latent variables are expressed as estimates of *X*_*t*_ and its first and second derivatives. The fixed factor loading of the first and second order derivatives of *X*_*t*_ are included in the loading matrix. GLLA is used to calculate approximate derivatives of a differential equation from repeated measures, and is defined as:

(3) $$Y=X^{D}W,$$

Where the matrix *Y* contains columns with the displacement *X*_*t*_ and its first and second order derivatives $\dot{X}$_*t*_ and $\overset{..}{X}$_*t*_, *X*^*D*^ is the time delay embedding matrix with dimension *D*, and *W* is the time related matrix of weights with *W* = *L*(*L*^*T*^*L*)^−1^ where *L* is the loading matrix and *L*^*T*^ is the transpose of the loading matrix.

The example below (Hu et al., 2014) demonstrates a TDE for *D* = 5 constructed from the original time series *X*_*pt*_, with *t* = 1, …, *T* observations for each individual *p* = 1, …, *N*.

(4) $$\begin{array}{l} X^{\left(5\right)}=\left(\begin{array}{ccccc} X_{\left(1,1\right)} & X_{\left(1,2\right)} & X_{\left(1,3\right)} & X_{\left(1,4\right)} & X_{\left(1,5\right)}\\ X_{\left(1,2\right)} & X_{\left(1,3\right)} & X_{\left(1,4\right)} & X_{\left(1,5\right)} & X_{\left(1,6\right)}\\ . & . & . & . & .\\ . & . & . & . & .\\ . & . & . & . & .\\ X_{\left(1,T-4\right)} & X_{\left(1,T-3\right)} & X_{\left(1,T-2\right)} & X_{\left(1,T-1\right)} & X_{\left(1,T\right)}\\ X_{\left(2,T-4\right)} & X_{\left(2,T-3\right)} & X_{\left(2,T-2\right)} & X_{\left(2,T-1\right)} & X_{\left(2,T\right)}\\ . & . & . & . & .\\ . & . & . & . & .\\ . & . & . & . & .\\ X_{\left(N,1\right)} & X_{\left(N,2\right)} & X_{\left(N,3\right)} & X_{\left(N,4\right)} & X_{\left(N,5\right)}\\ X_{\left(N,2\right)} & X_{\left(N,3\right)} & X_{\left(N,4\right)} & X_{\left(N,5\right)} & X_{\left(N,6\right)}\\ . & . & . & . & .\\ . & . & . & . & .\\ . & . & . & . & .\\ X_{\left(N,T-4\right)} & X_{\left(N,T-3\right)} & X_{\left(N,T-2\right)} & X_{\left(N,T-1\right)} & X_{\left(N,T\right)} \end{array}\right)\\ Y=\left(\begin{array}{ccc} X_{\left(1,3\right)} & {\dot{X}}_{\left(1,3\right)} & {\ddot{X}}_{\left(1,3\right)}\\ X_{\left(1,4\right)} & {\dot{X}}_{\left(1,4\right)} & {\ddot{X}}_{\left(1,4\right)}\\ . & . & .\\ . & . & .\\ . & . & .\\ X_{\left(1,T-2\right)} & {\dot{X}}_{\left(1,T-2\right)} & {\ddot{X}}_{\left(1,T-2\right)}\\ X_{\left(2,T-2\right)} & {\dot{X}}_{\left(2,T-2\right)} & {\ddot{X}}_{\left(2,T-2\right)}\\ . & . & .\\ . & . & .\\ X_{\left(N,3\right)} & {\dot{X}}_{\left(N,3\right)} & {\ddot{X}}_{\left(N,3\right)}\\ . & . & .\\ . & . & .\\ . & . & .\\ X_{\left(N,T-2\right)} & {\dot{X}}_{\left(N,T-2\right)} & {\ddot{X}}_{\left(N,T-2\right)} \end{array}\right)\\ L=\left(\begin{array}{ccc} 1 & -2\tau & {\left(-2\tau \right)}^{2}/2\\ 1 & -1\tau & {\left(-1\tau \right)}^{2}/2\\ 1 & 0 & 0\\ 1 & 1\tau & {\left(1\tau \right)}^{2}/2\\ 1 & 2\tau & {\left(2\tau \right)}^{2}/2 \end{array}\right) \end{array}$$

The *W* matrix can be calculated in R by using the gllaWMatrix() function provided in Boker et al. (2009). The disadvantage of GLLA is that it produces a large amount of bias in the estimated frequency parameter η due to correlated errors in the estimates of the derivatives (Deboeck, 2010). For this reason, Deboeck (2010) developed an alternative method, GOLD, in order to eliminate the problem of correlated errors. The introduced method is based on orthogonal polynomials, meaning that in this case the *W* matrix is constructed to be an orthogonal matrix.

### Sample and measures

#### Study design

Within a completed study on longitudinal student achievement (Hülür et al., 2011a,b), *N* = 112 German students from the beginning of 9th grade until the end of 10th grade completed a 120 min test battery on student achievement and working memory. Over a period of 2 years, students participated in 40 test sessions. Within each point of measurement, each working memory task was administered twice. At each measurement occasion different versions of the tasks were used. One of the working memory capacity tasks used in this intensive longitudinal study was a memory updating task (Oberauer et al., 2000, 2003; Schmiedek et al., 2009).

#### Memory updating task (MU)

Each MU consisted of eight items per measurement. The first two items consisted of three numbers and six mathematical operations, the middle four items of four numbers and eight operations, and the last two items of five numbers and 10 operations. The numbers within each item were presented for 4000 ms and disappeared after 500 ms. The arithmetic operations were displayed for 2000 ms for one half of the items and 1500 ms for the other half. The length of the interstimulus interval was 250 ms for all items. The working memory capacity data was scored using the proportion of correct answers at each point of measurement. The measurements were collected on a weekly basis so the observations were scaled by weeks.

#### Participants

The mean age of students at the beginning of the study was *M* = 14.7 (*SD* = 0.70), the proportion of female students was 64.3% (72 female and 40 male students).

### Analysis procedure

**Figure 1:**
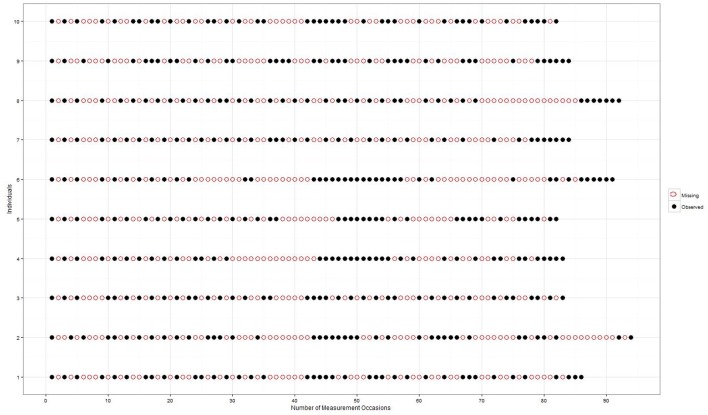
**Missing data pattern of 10 different individuals over 2 years in weeks**.

The main focus of the present analysis is whether there is an oscillation pattern in the memory updating (MU) performance of students as they progress from 9 to 10th grade, and how it differs between individuals. From visual inspection of the longitudinal data, it appears that there may be a non-linear change and a type of oscillatory pattern in the data over time. As the students participated in the study on unequally spaced measurement occasions, longitudinal observations of the MU task collected on 40 occasions were fit to the same time scale, with one measurement occasion per week. If the time elapsed between observations amounted to more than 1 week; missing values were inserted in the data set. Initially the students were tested every 2 weeks, but over the entire data collection period it was not possible to maintain this consistently. Hence, the amount of missing data was around 50%. Each individual has exactly 40 observations, but a different number of missing observations. The typical missing pattern of 10 students is depicted in Figure 1 over 97 weeks.

**Figure 2:**
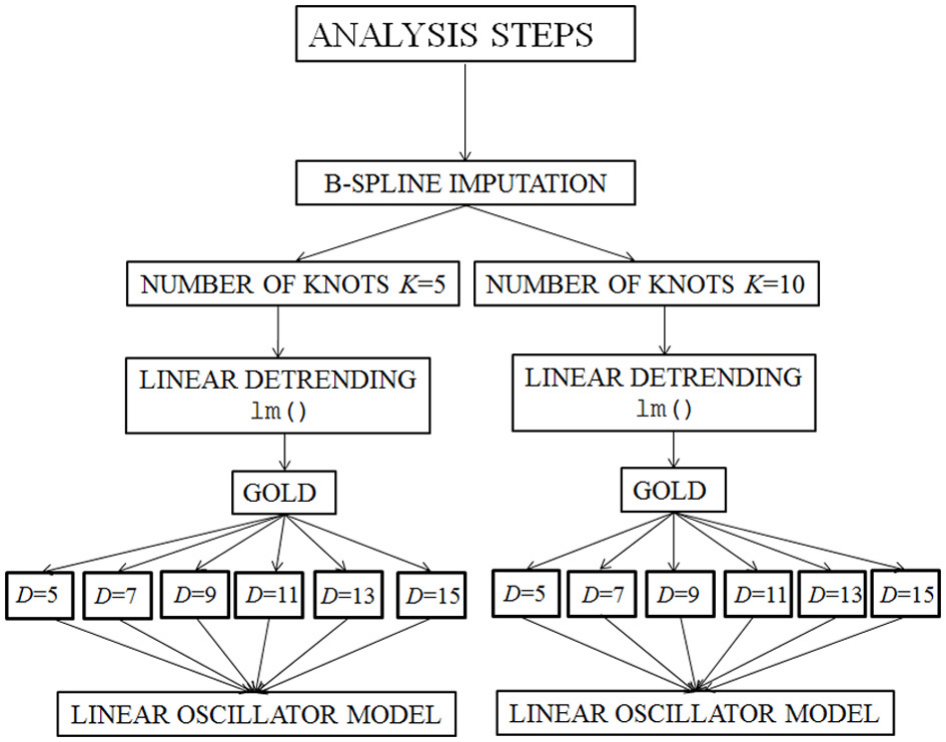
**Illustration of the applied method**. *K*, number of knots in the B-spline imputation; LM, lm() function; GOLD, Generalized Orthogonal Local Derivatives; *D*, time-delay embedding dimension.

The analysis of the data required several steps, illustrated in Figure 2 below. The applied techniques allow us to represent the individual differences within the linear mixed model.

By analyzing the data with the multilevel linear oscillator method, data was imputed by a B-spline imputation with a differing number of knots. A large amount of missing data requires a flexible imputation method. The result obtained from the spline imputation using the R splines package (Bates, 2011) showed slightly over-smoothed imputed data. For this reason we chose B-spline imputation as a missing data imputation method.

#### Missing data imputation

**Figure 3:**
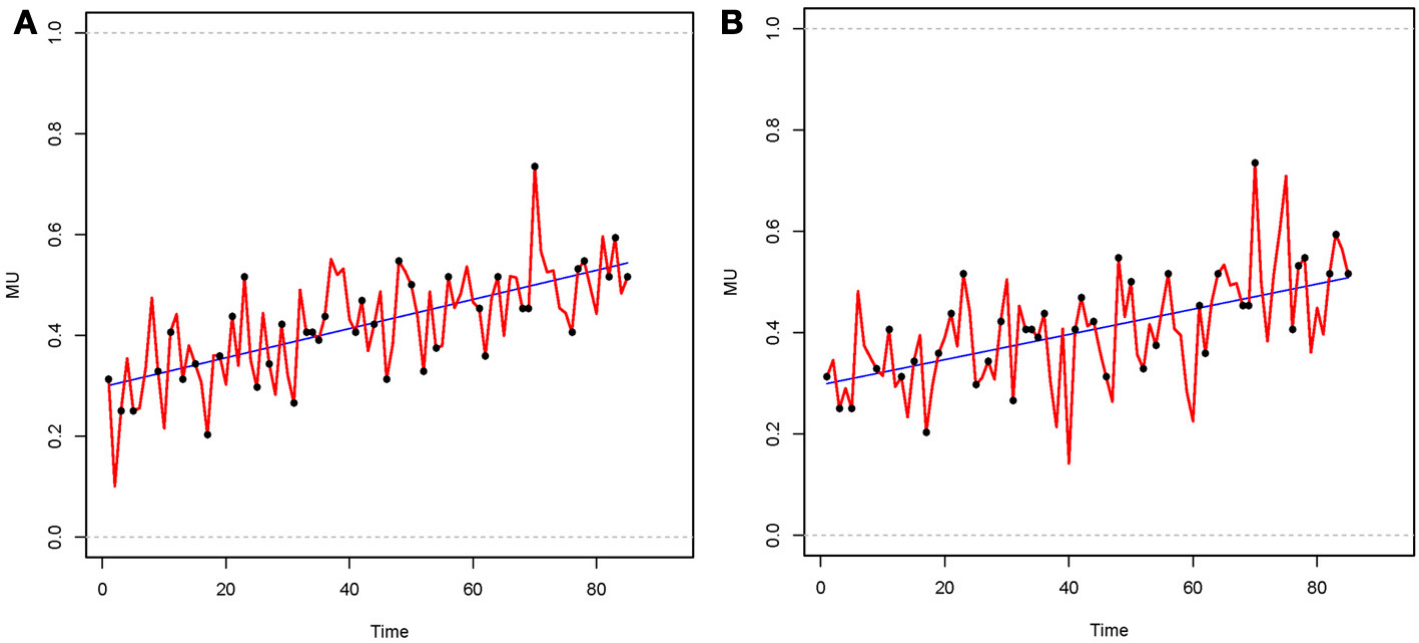
**B-spline imputed data of the same individual**. Black dots on the curve denote the observed data, while pieces between were imputed. **(A)** Number of knots *K* = 5; **(B)** Number of knots *K* = 10.

To handle the missing measurement points, a B-spline imputation was implemented in order to impute cyclic data (R code is given in Racine, 2011). A B-spline function is a piecewise polynomial function, with each of its pieces connected via knots (*K* is the number of knots). Schumaker (2007) and Wahba (1990) give a detailed introduction to B-spline imputation. To demonstrate the data imputation technique, Figure 3 below show data for a single individual imputed with the B-spline method.

In the first analysis step, a B-spline was estimated for each individual, based on their incomplete data set. In the next step, regression coefficients of the estimated B-spline were sampled, and missing values were imputed based on stochastic regression imputation (Little and Rubin, 2002). The number of measurement points for each individual result from implementation varied between a minimum of 77 and a maximum of 97. We applied the numbers of knots *K* = 5 and *K* = 10 in order to investigate how a different number of knots influences the obtained results. The number of knots has a direct influence on the variability of the imputed data. A small number of knots oversmoothes the data and removes the variability, whereas a big number of knots has the risk of a larger variability, which gives a biased representation of the data. For adequate results both cases should be chosen with necessary diligence. For this reason we provided a simulation study on the number of knots, to assess the accuracy of the applied B-spline imputation.

#### Linear detrending

Before estimating dynamics the analysis, we need to center the time series about their respective equilibrium, to do this we estimated residuals for each individual on a weekly basis by applying linear regression (Bisconti et al., 2004, 2006). Using R's R Development Core Team (2012) lm (linear models for fixed effects) function^1^. To linear detrend separately for each individual. Residuals, intercept and slope from the linear mixed effects model for each individual were estimated for each of the two B-spline imputation conditions (*K* = 5, *K* = 10) as

(5) $$Y_{pt}=I_{p}+S_{p}T_{p}+e_{pt},$$

Where *Y*_*pt*_ is the weekly achievement in MU of each person *p* on occasion *t*, *I*_*p*_ represents the initial level of each individual, *S*_*p*_ is the slope over time for each individual *p*, *T*_*p*_ indicates each person's observation point, and *e*_*pt*_ represents the normally distributed residual process.

The time elapsed between measurement point *t* and *t* – 1 was set at 1 week. For the estimating the dynamics we used the residuals, since dynamically patterned intraindividual variability is represented by residuals. According to Nesselroade (1990a) residuals contain information about intraindividual variability [*e*_*pt*_ from Equation (5)].

#### Constructing a time-delay embedding matrix

Using the residuals obtained from the lm() function, we constructed six candidate time-delay embedding matrices using dimensions *D* = 5, 7, 9, 11, 13, 15. The embedding dimension *D* indicates how many columns are time delayed in the matrix. The number of observation delays between columns, τ was set to one (i.e., each successive observation was used to construct an embedding matrix).

#### GOLD method

First and second order derivatives were estimated using GOLD for each of six candidate time delay embedded matrices (*D* = 5, 7, 9, 11, 13, 15). Each of the B-spline data sets were imputed using two different numbers of knots (*K* = 5 and *K* = 10). The main idea behind the GOLD method is to apply an orthogonal transformation of the time-delay embedding matrix to obtain least square estimates of derivatives of the time series. As mentioned in the introduction, GOLD produces uncorrelated errors in estimating derivatives, in contrast to GLLA.

#### Fitting the models

In the last step we applied a multilevel damped linear oscillator model (Model 1) and an extension of this model with predictors (Model 2). These models have been previously used in the context of modeling intraindividual variability in emotional well-being in widows (Bisconti et al., 2004). The models were applied to each of the six candidate time delay embedded matrices (*D* = 5, 7, 9, 11, 13, 15 and embedding interval τ = 1). In order to capture individual differences between participants in their ability to accomplish the MU task, the multilevel approach was implemented using the lmer() function in the R package lme4 (Bates et al., 2011).

Model 1 is defined as

(6) $$\begin{array}{l} {\overset{..}{X}}_{pt}=\eta_{p}X_{pt}+\zeta_{p}{\dot{X}}_{pt}+\upsilon_{pt},\\ \eta_{p}=\mu_{\eta }+u_{\eta p}, \end{array}$$

(7) $$\zeta_{P}\;=\mu_{\zeta }+u_{\zeta p.}$$

Where *X*_*pt*_ represents the observed scores for each individual *p* on each week *t*, in this case the residuals. $\dot{X}$_*pt*_ and $\overset{..}{X}$_*pt*_ are the first and second order derivatives, η_*p*_ and ζ_*p*_ are frequency and damping parameters that vary among all individuals, μ_η_ and μ_ζ_ are the mean values of the frequency and damping parameters, *u*_η*p*_ and *u*_ζ*p*_ are the individual effects of the frequency and damping parameters.

The relationship between the zero order *X*_*pt*_ and second order derivatives $\overset{..}{X}$_*pt*_ is represented by η_*p*_ parameter. This parameter is the major determiner of fluctuations in weekly achievement in the MU task. The same is true for damping parameter, with corresponding individual effects *u*_η*p*_ and *u*_ζ*p*_.

We investigated how well individual's parameters from the linear growth model Equation (5), predicted the frequency in intraindividual variability in Model 2 Equations (8)–(10). The multilevel damped linear oscillator in Model 2 was specified with individual-level predictors intercept *I*_*p*_ and slope *S*_*p*_. A similar approach was reported in Bisconti et al. (2006), where the pattern of the intraindividual variability was examined by predictors. Model 2 can be written:

(8) $${\overset{..}{X}}_{pt}=\eta_{p}X_{pt}+\zeta_{p}{\dot{X}}_{pt}+\upsilon_{pt},$$

(9) $$\eta_{p}=\mu_{\eta }+b_{01}I_{p}+b_{02}S_{p}+u_{\eta p},$$

(10) $$\zeta_{p}=\mu_{\zeta }+b_{11}I_{p}+b_{12}S_{p}+u_{\zeta p}.$$

Both Models 1 and 2 contain student-specific frequency and damping parameters, with random effects for the frequency and damping parameters. The predictors in Equations (9) and (10) describe the relationship between the derivatives and each individual's intercept (*I*_*p*_), as well as each individual's slope (*S*_*p*_) over all measurements. When individuals behave differently, person-specific values of intercept and slope can be used to predict the frequency η and damping ζ parameters.

The analysis steps were repeated 10 times, due to the imputation of missing data. We also calculated the period of oscillation λ_*p*_ Equation (2). As the data has been scaled by weeks, this formula represents the mean period of oscillation in units of weeks over all individuals.

## Simulation study

In an analysis pipeline with as many steps and choices as the current method, it is wise to question how well the method is recovering parameters of interest. In addition one can reasonably question how each of the analytic choices might affect results. In order to better understand how this method is performing for a data set with similar characteristics, we performed a small simulation study.

In order to explore how well the method works, we simulated 1000 data sets that describe fluctuations of *N* = 112 linear oscillators over a minimum of 77 and a maximum of 97 occasions. The pattern of the missing data in the simulated data set was defined to be identical to the original data of the longitudinal study on student achievement study. The known parameter values used to generate the data sets were chosen from results with the number of knots *K* = 10, dimension *D* = 11, frequency parameter η = −0.0245 (*SD* = 0.0001) and damping parameter ζ = 0 (*SD* = 3.74 × 10^(−10).^). Additionally, each of the 1000 simulated data sets was treated with the B-spline imputation method under four different conditions: *K* = 5, *K* = 8, *K* = 10, and *K* = 15. For each value of *K*, the imputed data were linearly detrended using the lm() function. The residuals were used to construct six candidate time delay embedding matrices with dimensions *D* = 5, 7, 9, 11, 13, 15, and embedding interval τ = 1. Subsequently, the GOLD method was used to estimate derivatives. The parameters of the multilevel linear oscillator model were estimated using Equations (6) and (7) (see the Supplementary Material for the R script). The procedure described above was repeatedly applied to the simulated data set with missing values 10 times for each value of *K* = 5, 8, 10, 15 and each dimension *D* = 5, 7, 9, 11, 13, 15. The results of 10 repetitions were combined by the mitools package. To assess the accuracy of the estimation we used following criteria:

1. Bias was calculated as the difference between the true parameter values and the means of sample parameter estimates over 1000 replications.
2. Root mean square error (RMSE) of the parameter estimates was calculated as the root mean squared difference between the sample parameter estimates and the true parameter value.

## Results

The results are based on memory updating data collected over 2 years on 40 measurement occasions, with *N* = 112 German students from the beginning of 9th grade until the end of 10th grade. After including occasions missing data, the number of measurement points varied from 77 to 97 per student. First, we present the B-spline imputation method with *K* = 5 and *K* = 10 knots to interpolate the missing data. Then the GOLD method to obtain estimates of the zeroth, first, and second derivatives from the B-spline imputed data. A linear mixed effects model was then used to estimate parameters of the linear oscillator (Bisconti et al., 2004, 2006). As described in the analysis section, we combined the results of 10 estimation procedures by using the MIcombine() function in the mitools package (Lumley, 2012) based on “Rubin's rules” of multiple imputation (Rubin, 1987).

We applied a damped linear oscillator model to the longitudinal data in order to explore the variability of the MU performance over a period of 2 years. First, the data were analyzed to detect an oscillation pattern, and then the frequency of the fluctuation was predicted by using the initial status of each individual. We only report values of the frequency parameter η and ζ, but neglect the interpretation of ζ. The significant negative value of η implies an oscillatory pattern.

Table 1 presents results of the B-spline imputed data fit by Model 1 Equation (6) and (7) for *K* = 5 and *K* = 10 over six distinct time-delay embedding dimensions *D*. Results show significant (α = 0.05) negative values of η over all embedding dimensions for *K* = 10. These values decrease when the dimension numbers increase. For *K* = 5, values of η were not significant for *D* = 5, but were significant with time delay embedding dimensions *D* ≥ 7. As shown in Table 1, the number of knots *K* influences the estimated values. The results of Model 1 with *K* = 10 for B-spline imputed data show a negative frequency parameter η. For *D* = 7 and *D* = 9, η appeared to be very similar: η_*D* = 7_ = −0.0269 (*SE* = 0.0016) and η_*D* = 9_ = −0.0261 (*SE* = 0.0010).

**Table 1:** **Estimated fixed effect of Parameter η using Model 1 applied on B-spline imputed data**.

**Model 1**	**GOLD-Function with B-SPLINE imputation**					
**LM**	***K* = 5**			***K* = 10**		
	***Est*.**	***SE***	***t***	***Est*.**	***SE***	***t***
**PARAMETER η (μ_η_)**						
*D* = 5	−0.0076	0.0041	−1.83	−0.0238	0.0026	−9.04
*D* = 7	−0.0089	0.0026	−3.41	−0.0269	0.0016	−16.69
*D* = 9	−0.0108	0.0012	−8.41	−0.0261	0.0010	−24.85
*D* = 11	−0.0098	0.0007	−12.43	−0.0247	0.0012	−19.67
*D* = 13	−0.0088	0.0007	−11.10	−0.0224	0.0007	−30.89
*D* = 15	−0.0081	0.0006	−13.33	−0.0212	0.0006	−30.91
**PARAMETER ζ (μ_ζ_)**						
*D* = 5	0.0020	0.0024	0.82	0.0007	0.0029	0.24
*D* = 7	0.0022	0.0019	1.17	−0.0008	0.0039	−0.20
*D* = 9	0.0012	0.0035	0.33	−0.0028	0.0026	−1.08
*D* = 11	0.0004	0.0023	0.18	0.0002	0.0019	0.14
*D* = 13	−0.0002	0.0017	−0.16	0.0000	0.0033	0.02
*D* = 15	−0.0037	0.0023	−1.63	0.0022	0.0021	1.04

*Total number of observations 8469 with a sample size of 112 individuals (which means that df > 100 for t-tests); D, number of time-delay embedding dimension; K, number of knots; LM,* lm() *function; Results are based on 10 times estimated values combined in one by the MIcombine() function; μ_η_ and μ_ζ_ represent the fixed effects of frequency and damping parameters*.

Table 2 contains the results of η predicted by intercept and slope for B-spline imputed data with *K* = 5 and *K* = 10. Estimated values differ from each other within the B-spline imputed data, both across the number of knots and across time-delay embedding dimensions *D*. As displayed in Table 2, the intercept is a significant predictor for frequency only for *K* = 10 for embedding dimensions *D* = 9 (*t* = 2.90), *D* = 11 (*t* = 3.24), *D* = 13 (*t* = 2.34), and *D* = 15 (*t* = 3.40). The positive relationship implies that an individual having a higher initial performance level at the MU task is associated with a slower frequency of fluctuation. The slope is significant in predicting a slower fluctuation for *K* = 5 with *D* = 11 and *D* = 15, and for *K* = 10 across all dimensions except for *D* = 11 and *D* = 15. This means that individuals who fluctuate more often have less improvement at the MU task over time. Since we specified Model 1 and Model 2 as multilevel models, we also calculated the random effects of each parameter. Random effects were estimated as nonzero for embedding dimension *D* = 11, *D* = 13, and *D* = 15.

**Table 2:** **Estimated fixed effect of parameter η predicted by intercept and slope using Model 2 applied on B-spline imputed data**.

**Model 2**	**GOLD-Function with B-SPLINE Imputation**					
**LM**	***K* = 5**			***K* = 10**		
	***Est*.**	***SE***	***t***	***Est*.**	***SE***	***t***
**PARAMETER η (μ_η_)**						
*D* = 5	0.0256	0.0076	3.37	−0.0374	0.0107	−3.48
*D* = 7	−0.0142	0.0050	−2.83	−0.0343	0.0050	−6.81
*D* = 9	−0.0171	0.0035	−4.84	−0.0377	0.0039	−9.54
*D* = 11	−0.0133	0.0029	−4.45	−0.0361	0.0038	−9.29
*D* = 13	−0.0121	0.0014	−8.13	−0.0319	0.0035	−9.05
*D* = 15	−0.0107	0.0013	−7.83	−0.0295	0.0024	−11.80
**η: *I*_*p*_ (*b*_01_)**						
*D* = 5	0.0083	0.0291	0.28	0.0232	0.0240	0.96
*D* = 7	0.0107	0.0099	1.07	0.0112	0.0108	1.03
*D* = 9	0.0136	0.0078	1.73	0.0223	0.0077	2.90
*D* = 11	0.0051	0.0064	0.80	0.0225	0.0069	3.24
*D* = 13	0.0040	0.0035	1.13	0.0187	0.0079	2.34
*D* = 15	0.0033	0.0031	1.06	0.0163	0.0047	3.40
**η:*S*_*p*_ (*b*_02_)**						
*D* = 5	0.7851	1.7936	0.43	2.5863	0.9274	2.78
*D* = 7	0.5483	0.4909	1.11	1.7146	0.7441	2.30
*D* = 9	0.3256	0.5134	0.63	1.4729	0.5495	2.68
*D* = 11	0.7839	0.2618	2.99	1.0904	0.9765	1.11
*D* = 13	0.9825	0.5693	1.72	1.0610	0.4530	2.34
*D* = 15	0.7666	0.2816	2.72	0.8281	0.4493	1.84

*Total number of observations 8469 with a sample size of 112 individuals (which means that df > 100 for t-tests). D, number of time-delay embedding dimension; K, number of knots; LM,* lm() *function. Results are based on 10 times estimated values combined in one by MIcombine() function. μ_η_ and μ_ζ_ represent the fixed effects of frequency parameter; I_p_, Intercept; S_p_, Slope with regression coefficients b_01_ and b_02_*.

**Figure 4:**
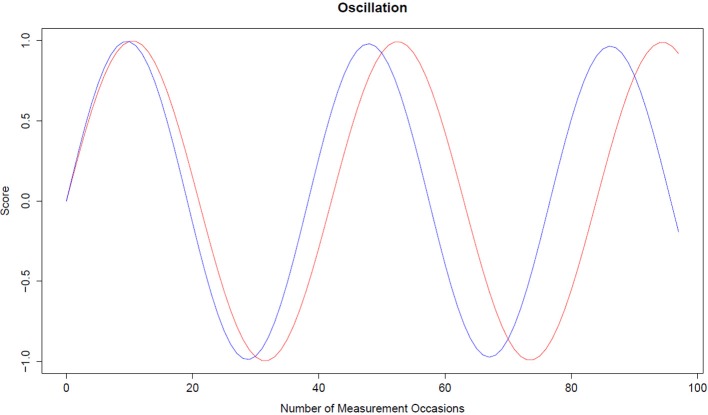
**Oscillation curve**. The red line represents an oscillation for *D* = 11 and *K* = 10 with η = −0.0224, ζ = −0.0002, and λ = 40. The blue line represents an oscillation for *D* = 7 and *K* = 10 with η = −0.0269, ζ = −0.0008, and λ = 38.3.

Using the fixed effects we transformed the estimated values into a period of oscillation. We report the mean period of oscillation for Model 1. Model 2 describes the dependency of the oscillation period from the covariates intercept and slope. The period of oscillation lasts between 38 and 40 weeks and might indicate that MU performance is connected to the school routine. One oscillation period begins at the beginning of the school year and ends at the end of the school year. Fluctuations of working memory performance from day to day were also investigated by Brose et al. (2012). The number of weeks for one competed cycle λ Equation (2) for data imputed with B-spline imputation with *K* = 10 and *D* = 5 was equal to λ = 40 weeks, calculated from Model 1. For *D* = 7, the period was λ = 38.3 (Figure 4), *D* = 9, λ = 38.8, *D* = 11, and *D* = 13 the number of weeks for one oscillation was approximately 40 weeks (Figure 4). Figure 4 depicts an oscillation (sinus curve) for *D* = 7 and *D* = 13. The number of weeks needed for one cycle depends not only on the time-delay dimension *D*, but also on the number of knots *K* within the B-spline imputation method.

For Model 2 we predicted the frequency parameter and the oscillation period length by setting the covariates intercept and slope at their mean, minimum and maximum values. The mean value of the intercept over 112 individuals was *M* = 0.443 (*SD* = 0.167), whereas the mean value of the slope was *M* = 0.0015 (*SD* = 0.0018). We predicted the frequency for B-spline imputed data with *K* = 10 and *D* = 13. The predicted frequency for a student with a mean intercept and a mean slope was η = −0.0124 with an oscillation period of 56 weeks. At the value of one standard deviation below the mean intercept (0.2760) and mean slope (−0.0002) the predicted frequency was −0.0175 with an oscillation period equal to 47 weeks. At one standard deviation above the mean intercept (0.6100) and mean slope (0.0034) the predicted frequency was η = −0.0073 with a period of oscillation equal to 73 weeks. The period of oscillation is influenced by the frequency Equation (2), i.e., the smaller the frequency the shorter the period. The correlation between intercept and slope was *r* = −0.074. The relationship between the frequency parameter and initial performance level was positive. Since the frequency parameter value is negative, this means that the smaller the frequency the lower the initial level. Therefore, the smaller the frequency parameter (with η < 0) the shorter the length of oscillation period. The relationship between frequency and improvement on the MU task over time is positive. This can be interpreted as the less the improvement over time the lower the frequency value of students, and the more often they fluctuate.

We implemented the LDE model in WinBUGS by applying a Bayesian approach, whereas the GOLD Method was implemented in R by using the GOLD function. The latter method requires imputation of missing values in the longitudinal data. An LDE model in a Bayesian framework was implemented using the R2WinBUGS package (Bayesian inference using Gibbs Sampling, Lunn et al., 2000; Spiegelhalter et al., 2003). In contrast to the LDE model in OpenMx (Boker et al., 2010b), LDE in WinBUGS allows for individual differences, as WinBUGS incorporates the hierarchical structure of the data. The idea behind the usage of WinBUGS was to analyze the data in one-step within the multilevel approach, without imputing the missing data. The data contained missing values, but these values were not imputed by using a B-spline. We chose the number of iterations to be 10,000, with 5000 burn-in iterations. We used the mean of the sampled values of the posterior distribution as an estimator of the parameter of interest. In the empirical illustration of this paper, we verified the convergence by visually monitoring the trace plots. Brooks and Gelman (1998) suggested a number of convergence tests, emphasizing that a single-chain diagnostic depends on the starting points of the simulation. The LDE model was fit to the same data set as the GOLD model. As shown in Table 3, results obtained for embedding dimension *D* = 11 reveal a larger value for the frequency parameter η, which also implies less fluctuation in the data and a longer oscillation period.

**Table 3:** **Estimated fixed effect of parameter η and ζ using LDE (WinBUGS) and GOLD**.

	**Embedding dimension *D* = 11**								
	**LDE**			**GOLD *K* = 5**			**GOLD *K* = 10**		
	***Est*.**	***SE***	***t***	***Est*.**	***SE***	***t***	***Est*.**	***SE***	***t***
**MODEL 1**									
η (μ_η_)	−0.0611	0.0074	−8.21	−0.0098	0.0007	−12.43	−0.0247	0.0012	−19.67
ζ (μ_ζ_)	−0.0040	0.0143	−0.28	0.0004	0.0023	0.18	0.0002	0.0019	0.14
**MODEL 2**									
η (μ_η_)	−0.0523	0.0095	−5.70	−0.0133	0.0029	−4.45	−0.0361	0.0038	−9.29
η: *I* (*b*_01_)	0.0042	0.0250	0.16	0.0051	0.0064	0.80	0.0225	0.0069	3.24
η: *S*_*p*_(*b*_02_)	−0.0006	0.0002	−3.12	0.7839	0.2618	2.99	1.0904	0.9765	1.11

*D, number of time-delay embedding dimension; K, number of knots. μ_η_ and μ_ζ_ represent the fixed effects of frequency and damping parameters; I_p_, Intercept; S_p_, Slope with regression coefficients b_01_ and b_02_*.

The parameter estimation based on LDE in WinBUGS has several practical issues. First, the algorithm failed to converge with the increasing size of the time-delay embedding dimension. For example, values of dimension *D* = 13 and higher caused the algorithm to crash. Second, further research is needed to identify appropriate initial values.

In order to verify that the estimated results are consistent, the jackknife technique was applied. The jackknife technique (Cameron and Trivedi, 2005) is a resampling method, and is used to provide more information about uncertainty in estimates. We calculated the jackknife estimation of the standard error of an estimate $\hat{\theta }$ that captures the variability between subsamples.

(11) $$SE_{JK}\left[\hat{\theta }\right]={\left[\frac{N-1}{N}\sum_{p\;=\;1}^{N}{\left({\hat{\theta }}_{\left(-p\right)}-\bar{\hat{\text{​​}\theta }}\right)}^{2}\right]}^{1/2}$$

*N* jackknife replication estimates $\hat{\theta }$_(−*p*)_ are obtained by deleting observations of the *p*th subject for *p* = 1, …, *N*, then recomputing the estimates from a reduced sample size (*N* − 1), where $\hat{\theta }$_*N*_ is an estimate computed over the original sample size *N*. $\bar{\hat{\theta }}=\frac{1}{N}\sum_{p\;=\;1}^{N}{\hat{\theta }}_{\left(-p\right)}$ is an average across *N* jackknife replication estimates $\hat{\theta }$_(−*p*)_.

(12) $$\begin{array}{cc} t_{JK}\left[\hat{\theta }\right]=\frac{{\hat{\theta }}_{\left(-p\right)}-\;\bar{\hat{\text{​​}\theta }}}{{\left[\frac{N-1}{N}\sum_{p\;=\;1}^{N}{\left({\hat{\theta }}_{\left(-p\right)}-\bar{\hat{\theta }}\right)}^{2}\right]}^{1/2}} & \left(12\right) \end{array}$$

Table 4 shows results obtained from jackknife inference, for dimension *D* = 13 with *K* = 5, and *K* = 10. Results of the full sample were obtained from a single implementation of the analysis steps. Estimates of the standard error and *t*-value obtained from jackknife inference differ very little from the full sample estimates, indicating consistent results.

**Table 4:** **Results for the full sample (*N* = 112) and jackknife sample, with number of knots *K* = 5 and dimension *D* = 13**.

**D = 13**	**B-spline**							
**LM**	***K* = 5**				***K* = 10**			
	***FS.SE***	***FS.t***	***JK.SE***	***JK.t***	***FS.SE***	***FS.t***	***JK.SE***	***JK.t***
**MODEL 1**								
η (μ_η_)	0.0011	−7.00	0.0010	−7.61	0.0016	−14.32	0.0016	−14.33
ζ (μ_ζ_)	0.0052	−0.45	0.0055	−0.42	0.0045	−0.35	0.0041	−0.39
**MODEL 2**								
η (μ_η_)	0.0034	−4.24	0.0030	−4.82	0.0047	−8.44	0.0049	−8.12
ζ (μ_ζ_)	0.0165	1.60	0.0119	2.22	0.0132	2.49	0.0145	2.26
η: *I*_*p*_(*b*_01_)	0.0071	1.39	0.0057	1.72	0.0095	3.50	0.0096	3.45
η: *S*_*p*_(*b*_02_)	0.4681	2.98	0.4758	2.93	0.7285	2.13	0.7344	2.11

*Results of one implementation of the analysis for Model 1 and Model 2. FS, Full sample; JK, Jackknife sample. μ_η_ and μ_ζ_ represent the fixed effects of frequency and damping parameters; I_p_, Intercept and S_p_, Slope with regression coefficients b_01_ and b_02_*.

## Results of the simulation study

Results presented in Table 5 contain the true value η, bias, RMSE and means of estimates from 1000 data points, with the estimation procedure applied 10 times repeatedly to the data due to the imputation of missing values. The mean values of 10 estimation repetitions were combined in one via the MIcombine() function. Each of the generated data points contained a sample size *N* = 112 (sampling interval was set at 1 week) with the different number of missing values (from 77 to 97) and exactly the same number of 40 observations. The initial values for the generation of the data were calculated using *K* = 10. As expected, the number of knots and the value of the embedding dimension have a strong impact on the simulation results. For the number of knots *K* = 15, the bias becomes smaller with the increasing size of the time-delay embedding dimension *D*. As shown in Table 4 for *D* = 5 bias = −0.0289, whereas for *D* = 15 bias is equal to −0.0041. The best estimates (i.e., with the smallest values of bias and RMSE) were obtained for *K* = 8. The estimates for *K* = 5 were underestimated, while for *K* = 10 and *K* = 15 the values were overestimated. The calculated RMSE values are very close to the bias values, which indicate that the standard deviation of the estimates is close to zero. The reported results show that the number of knots used in the B-spline influence accuracy more than the dimension *D*. This simulation examined the linear oscillator model, focusing on the previously applied B-spline imputation method. The results of this simulation study indicate that for *K* = 5, *K* = 10, and *K* = 15, small embedding dimension values give a poor reflection of the true value.

**Table 5:** **Mean estimates, Bias and RMSE of the linear oscillator parameter η for *K* = 5, *K* = 8, *K* = 10, and *K* = 15 across all dimension conditions**.

**True η_μη_ = −0.0245**	***K* = 5**			***K* = 8**			***K* = 10**			***K* = 15**		
	**Mean(η)**	**BIAS**	**RMSE**	**Mean(η)**	**BIAS**	**RMSE**	**Mean(η)**	**BIAS**	**RMSE**	**Mean(η)**	**BIAS**	**RMSE**
*D* = 5	−0.0156	0.0089	0.0090	−0.0244	0.0001	0.0013	−0.0288	−0.0042	0.0044	−0.0535	−0.0289	0.0290
*D* = 7	−0.0161	0.0084	0.0085	−0.0251	−0.0005	0.0011	−0.0290	−0.0044	0.0045	−0.0473	−0.0227	0.0228
*D* = 9	−0.0160	0.0085	0.0086	−0.0254	−0.0008	0.0012	−0.0285	−0.0039	0.0040	−0.0416	−0.0170	0.0170
*D* = 11	−0.0168	0.0077	0.0078	−0.0253	−0.0007	0.0011	−0.0279	−0.0033	0.0034	−0.0364	−0.0118	0.0119
*D* = 13	−0.0174	0.0071	0.0071	−0.0254	−0.0008	0.0012	−0.0277	−0.0031	0.0033	−0.0320	−0.0074	0.0075
*D* = 15	−0.0180	0.0065	0.0065	−0.0259	−0.0013	0.0016	−0.0278	−0.0032	0.0034	−0.0286	−0.0041	0.0042

*Combined means of the repeatedly calculated estimates from 1000 simulated data sets were presented. K is the number of knots in B-spline imputation and D the number of time-delay dimensions. η_μ_ represents the fixed effect of frequency parameter*.

## Discussion

In this paper, we investigated intraindividual variability in memory updating performance using dynamical systems analysis applied to a longitudinal study over a period of 2 years with. This paper adds findings for estimating the parameters of a damped linear oscillator model for applications with sparse measurements in time.

First, we introduced the B-spline imputation method for cyclic data to account for missing data. Subsequently, we analyzed the data by using a multilevel linear oscillator model, which is represented by a second-order differential equation. The GOLD Method estimated the derivatives and parameters of interest that represent the intraindividual variability, such as the frequency parameter (i.e., change in fluctuation). Brose et al. (2012) captured trends in cognitive performance by spline smoothing while investigating daily variability in working memory. Nesselroade (1990a) analyzed differentiation between short-term fluctuations and changes over long periods of time. Intraindividual variability represents short-term changes or fluctuations, whereas intraindividual change implies long-term changes. Intraindividual variability was captured using the residuals of each person's performance on weekly assessments of working memory capacity. Visual examination of the data showed that there are person-specific differences in their trajectories. This means that analysis based on the overall mean trajectory represents inadequate individual change over time. Due to individual differences, we applied multilevel modeling in order to account for each individual's frequency and damping parameter. Before fitting the multilevel linear oscillator model, we calculated derivatives from B-spline imputed data using GOLD in order to estimate zeroth, first and second order derivatives from a time delay embedding matrix. The values of lag between observations τ in the embedded matrix, and the value of embedding dimension *D*, have an impact on estimates of derivatives (Boker and Nesselroade, 2002; Hu et al., 2014). They showed that the Akaike Information Criterion (AIC) and −2 log likelihood (−2LL) based criteria, recommended by Boker and Nesselroade (2002), are not reliable to select *D*, and suggested instead a new rule that uses the estimated values of the frequency parameter η. The recommended procedure to find the optimal *D* is to plot estimated values of η as a function of parameter *D*. The first value of *D* that results in a stable estimation of η is the optimal choice. In our case this criterion was not appropriate, since we did not obtain reliable stable values of estimated η. In this study, the time delay embedding matrix based on residuals was used, and grouped by student ID numbers, with dimension *D* = 5, 7, 9, 11, 13, 15, and τ = 1. This means that the time-delay ranged between 5, 7, 9, 11, 13, and 15 columns of embedded matrices constructed from repeated measures set at 1 week. Students' weekly performance in the MU task was modeled so as to estimate frequency, meaning how slowly or quickly each participant fluctuates over a certain period of time. To test whether each person showed oscillatory patterns in their weekly performance in the MU task, the dynamical systems oscillator model was applied to the residuals of each individual at each measurement point. Next, we specified a multilevel mixed effects model to investigate whether the frequency and damping parameters might be predicted by the initial status and rate of change. A significant negative value of η and significant fixed effects of intercept indicate that individuals with high initial performance values oscillate more slowly in their weekly achievement in the MU task. Furthermore, individuals with lower initial performance levels showed greater variability than those with higher performance levels. These findings are supported by the literature, in which intraindividual variability has been associated with inconsistency in cognitive performance (Hultsch et al., 2000; Ram et al., 2005). Hultsch et al. (2000) found that healthy adults with lower cognitive performance level show more inconsistency in their performance than individuals with higher cognitive performance level. Ram et al. (2005) termed week-to-week intraindividual variability as random noise and suggested that individuals with higher random noise levels perform lower on intelligence tests. The estimation of dynamical systems analyses using differential equations has become more popular among researchers for describing intraindividual variability, particularly since intraindividual variability is considered to be a cyclic process. Findings over all models and knots within imputations showed an oscillation pattern in the data. For number of knots *K* = 5 and *K* = 10 the mean number of weeks for one completed oscillation ranged from approximately 40 weeks, which might be associated with the end of a school year and start of summer break.

To better understand the sensitivity of estimates toward the number of knots *K* in the B-spline method, a simulation study was conducted based on the examination of bias and RMSE values of simulated data. The data sets were simulated using the damped linear oscillator model. The initial values for frequency η and damping ζ parameter were calculated from B-spline imputed data with *K* = 10 within the GOLD method in Model 1 for time-delay embedding dimension *D* = 11. The simulated data set included missing values with the same pattern as the original data. In the simulation study the best estimates were obtained by using number of knots *K* = 8. In this case, the difference between the true value and the mean estimate was smaller than using *K* = 10, *K* = 15, or *K* = 5.

The realization of the LDE model in WinBUGS showed implementation issues while using a higher order of time delay embedding dimension, due to an error in the WinBUGS application. Another issue of the implementation was the difficulty in choosing initial values for the algorithm. The GOLD method also had algorithmic limitations, as it is limited to the univariate case. Another concern is related to the B-spline imputation. In general using a large knot number *K* creates a large amount of noise in the data, whereas a small value of *K* over-smooths the trajectory. There is more research needed to explore how to select the optimal number of knots for the B-spline imputation.

### Conflict of interest statement

The authors declare that the research was conducted in the absence of any commercial or financial relationships that could be construed as a potential conflict of interest.

## Footnotes

^1^At the same time we applied the lme (linear models for mixed effects) function in order to estimate person-specific intercepts, slopes and residuals. Results obtained from lme() detrending do not differ from the lm() results, for both numbers of knots *K* = 5 or *K* = 10 in the B-spline imputation.
