# Supplementary material for: Dynamical systems analysis applied to working memory data
Source: Front Psychol. 2014 Jul 3;5:687. doi: 10.3389/fpsyg.2014.00687 (PMC4080465; doi:10.3389/fpsyg.2014.00687)
Supplement: Supplementary file 1 [file DataSheet1.DOCX]

APPENDIX

**Script for estimation simulated data using B-spline imputation, linear detrending, and GOLD method**

# Missing data treatment: B-spline

dat <- data.frame(dat)

dat$splinedmu <- NA

splinedmu <- rep(NA,length(dat$mu))

studentIDs <- unique(dat$id)

for( tID in studentIDs ){

tdat <- dat[dat$id==tID,]

tmin <- min(tdat$session[!is.na(tdat$mu)])

tmax <- max(tdat$session[!is.na(tdat$mu)])

tn <- length(tdat$session)

# Imputation using B splines

if (md[[1]] == "bspline-imp" ){

fm1 <- lm( mu ~ bs( session, df=md[[2]]) , data = tdat)

newx <- tmin:tmax

# Sampling of regression coefficients (TRUE or FALSE)

if ( md[[3]] ){

cf <- coef(fm1)

cfnew <- mvrnorm( 1 , mu = cf , Sigma = vcov(fm1) )

fm1$coefficients <- cfnew

}

# Prediction using B-splines

newy <- predict( fm1 , data.frame( session= tmin:tmax) )

# Addition of noise into regression imputation

newy <- newy + rnorm( length(newy) , sd = summary(fm1)$sigma )

dat$splinedmu[ dat$id == tID ] <- dat$mu[ dat$id == tID ]

ind <- is.na( dat$mu )

ind2 <- is.na( tdat$mu )

dat[ ind & ( dat$id == tID ) , "splinedmu" ] <- newy[ ind2 ]

}

print( paste( name.gold , tID) )

flush.console()

}

dat <- dat[ , c( "id","session" , "mu" , "splinedmu" ) ]

# Detrending the data using lm()

if ( detrend == "lm" ){

datNA_mu <-dat[!is.na(dat$splinedmu),]

datNA_mu$SResMu <- NA

datNA_mu$Slo <- datNA_mu$Int <- NA

for (tID in studentIDs){

Lmu <- lm( splinedmu ~ session, data = datNA_mu[ datNA_mu$id == tID , ] )

ind <- which( datNA_mu$id == tID )

datNA_mu$Int[ind] <- coef(Lmu)[1]

datNA_mu$Slo[ind] <- coef(Lmu)[2]

datNA_mu$SResMu[ind] <- resid( Lmu )

}

tDataFrame <- datNA_mu

}

write.csv2(tDataFrame, file.path( pf1sdd , paste( name.gold , "__DETRENDED-DATA.csv" ,sep="") ) , quote=F , row.names=F , na ="" )

# GOLD analysis

# Set constants for embedding dimensions and create the embedded data matrix.

theDeltaT <- 1

theTau <- 1

tEmbeddedData <- gllaEmbed(tDataFrame$SResMu, embed=embedD, tau=theTau, groupby=tDataFrame$id, label="X", idColumn=TRUE)

head(tEmbeddedData)

goldData <- matrix(NA, dim(tEmbeddedData)[1], 4)

dimnames(goldData) <- list(NULL, c("ID", "X", "dX", "d2X"))

goldData[,1] <- tEmbeddedData[,1]

wMatrix <- GOLDW(seq(0, theTau*(embedD-1), by=theTau),2)

goldData[,2:4] <- tEmbeddedData[,2:(1+embedD)]%*%wMatrix

head(goldData)

filterCenter <- floor((embedD + 1)/2)

for(tID in unique(tDataFrame$id)) {

covIndex <- c(filterCenter:(filterCenter-1+length(goldData[goldData[,1]==tID,1])))

}

goldFrame <- data.frame(goldData)

write.table( goldFrame, file.path( pf1sdd , paste( name.gold , "__GOLD-DATA.dat" ,sep="")), quote=F , row.names=F , na ="." )

# Mixed Effects Analysis

dfr <- NULL

ss <- 0

goldFrame.temp <- goldFrame[ goldFrame$ID != ss , ]

head(goldFrame.temp)

# GOLD model with random effects with lmer ()

# Model M1

mod.name <- "M1"

mod <- lmer( d2X ~ X + dX + ( 0 + X | ID ) + ( 0+dX | ID ) , data = goldFrame.temp )

summary(mod)

dfrtt <- summary(mod)

dfr.tt <- data.frame( "parameter" = rownames(coef(dfrtt)) , coef(dfrtt) )

vc <- VarCorr(mod)

vc <- c( unlist(vc) , attr(vc,"sc")^2)

dfr.tt2 <- data.frame("parameter" = c("var.X", "var.dX" , "var.resid"), vc , NA, NA)

colnames(dfr.tt2) <- colnames(dfr.tt )

dfr.tt <- rbind( dfr.tt , dfr.tt2 )

dfr.tt <- data.frame( "subject.elim" = ss , "name.gold" = name.gold , "missing" = md[[1]] , "missing.df" = md[[2]] , "missing.regr" = md[[3]] , "detrend" = detrend , "embedD" = embedD , "model" = mod.name , dfr.tt )

dfr <- rbind( dfr , dfr.tt )

print(mod.name) ; flush.console()

write.csv2( dfr, file.path( pf1sdd , paste( name.gold , "__LMER-RESULTS.csv" ,sep="") ) , quote=F , row.names=F , na ="" )

} # Number of imputations

} # Embedding dimension

} # Number of knots

} # datalist
